# Author Correction: Universal mechanical exfoliation of large-area 2D crystals

**DOI:** 10.1038/s41467-020-16733-4

**Published:** 2020-06-05

**Authors:** Yuan Huang, Yu-Hao Pan, Rong Yang, Li-Hong Bao, Lei Meng, Hai-Lan Luo, Yong-Qing Cai, Guo-Dong Liu, Wen-Juan Zhao, Zhang Zhou, Liang-Mei Wu, Zhi-Li Zhu, Ming Huang, Li-Wei Liu, Lei Liu, Peng Cheng, Ke-Hui Wu, Shi-Bing Tian, Chang-Zhi Gu, You-Guo Shi, Yan-Feng Guo, Zhi Gang Cheng, Jiang-Ping Hu, Lin Zhao, Guan-Hua Yang, Eli Sutter, Peter Sutter, Ye-Liang Wang, Wei Ji, Xing-Jiang Zhou, Hong-Jun Gao

**Affiliations:** 1 Institute of Physics, Chinese Academy of Sciences, 100190 Beijing, China; 2 Songshan Lake Materials Laboratory, 523808 Dongguan, China; 3 Department of Physics and Beijing Key Laboratory of Optoelectronic Functional Materials & Micro-Nano Devices, Renmin University of China, 100872 Beijing, China; 4 School of Materials Science and Engineering, Ulsan National Institute of Science and Technology (UNIST), Ulsan, 44919 Republic of Korea; 5 School of Information and Electronics, MIIT Key Laboratory for Low-Dimensional Quantum Structure and Devices, Beijing Institute of Technology, 100081 Beijing, China; 6 College of Engineering, Peking University, 100871 Beijing, China; 7 School of Physical Science and Technology, Shanghai Tech University, 201210 Shanghai, China; 8 University of Chinese Academy of Sciences, 100049 Beijing, China; 9 Institute of Microelectronics of Chinese Academy of Sciences, 100029 Beijing, China; 10 Department of Mechanical and Materials Engineering, University of Nebraska—Lincoln, Lincoln, NE 68588 United States; 11 Department of Electrical and Computer Engineering, University of Nebraska—Lincoln, Lincoln, NE 68588 United States

**Keywords:** Condensed-matter physics, Materials for devices, Two-dimensional materials

Correction to: *Nature Communications* 10.1038/s41467-020-16266-w, published online 15 May 2020.

The original version of this Article contained errors in the author affiliations.

Ye-Liang Wang was incorrectly associated with the School of Materials Science and Engineering, Ulsan National Institute of Science and Technology (UNIST), Ulsan 44919, Republic of Korea.

Wei Ji was incorrectly associated with the Songshan Lake Materials Laboratory, 523808 Dongguan, China.

These have now been corrected in both the PDF and HTML versions of the Article.

