# Supplementary material for: Universal mechanical exfoliation of large-area 2D crystals
Source: Nat Commun. 2020 May 15;11:2453. doi: 10.1038/s41467-020-16266-w (PMC7228924; doi:10.1038/s41467-020-16266-w)
Supplement: Supplementary file 1 — Supplementary Information [file 41467_2020_16266_MOESM1_ESM.pdf]

Supplementary Information for

Universal mechanical exfoliation of large-area 2D crystals

Yuan Huang[†], Yu-Hao Pan[†], Rong Yang[†], Li-Hong Bao, Lei Meng, Hai-Lan Luo, Yong-Qing Cai, Guo-Dong Liu, Wen-Juan Zhao, Zhang Zhou, Liang-Mei Wu, Zhi-Li Zhu, Ming Huang, Li-Wei Liu, Lei Liu, Peng Cheng, Ke-Hui Wu, Shi-Bing Tian, Chang-Zhi Gu, You-Guo Shi, Yan-Feng Guo, Zhi Gang Cheng, Jiang-Ping Hu, Lin Zhao, Guan-Hua Yang, Eli Sutter, Peter Sutter[*], Ye-Liang Wang, Wei Ji[*], Xing-Jiang Zhou[*], and Hong-Jun Gao[*]

[†]These authors contributed equally to this work.

*Correspondence to: psutter@unl.edu (P.S.); wji@ruc.edu.cn (W.J.); xjzhou@iphy.ac.cn (X.J.Z.); hjgao@iphy.ac.cn (H.J.G.)

Supplementary Figures

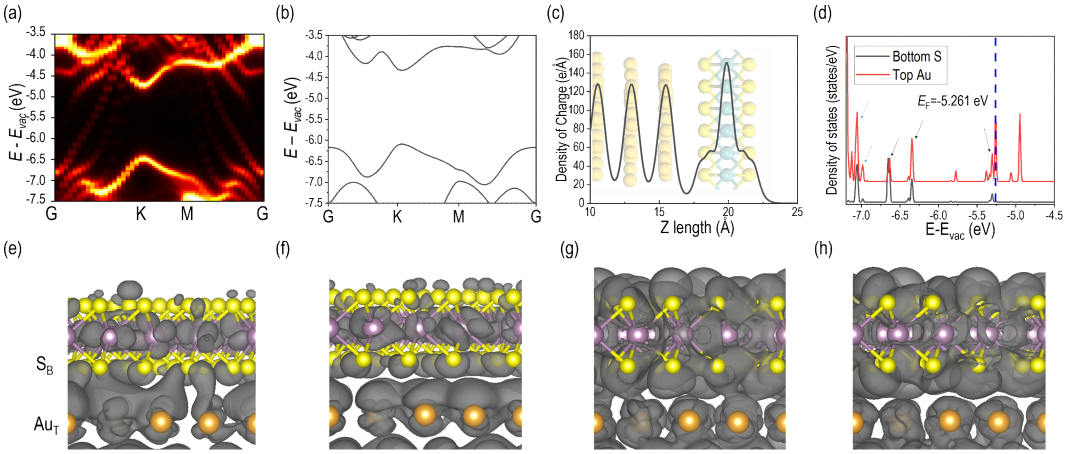

Supplementary Figure 1. Electronic structures of an Au (111) supported monolayer MoS_2 (1L-MoS_2), a typical 2D material, interface. Here, MoS_2 was chosen in consideration of that it has a moderate $R_{LA/IL}$ of 1.51 and group 16 (VIA) elements are the major portion of non-metal elements in all considered 2D layers. The vacuum energy was set as energy zero in both bandstructures and DOS plots. **(a)** Unfolded bandstructure of the interface, which was calculated using the KPROJ program based on the $\boldsymbol{k}$-projection method[1, 2]. **(b)** Bandstructure of freestanding 1L-MoS_2. The bandgap of 1.76 eV and the shape of valence (VB) and conduction (CB) bands remain nearly unchanged after the free-standing layer in contact with the Au substrate while the positions of VB and CB shift downward by ~0.5 eV. This shift is a result of vertical electric dipole moments (~0.75 e·Å) formed at the interface upon contact, which were illustrated by the DCD in Fig. 1e and a layer-averaged line profile associated with total electronic charge density, as plotted in **(c)**. Dipole moment $\boldsymbol{p}$ here is defined as $\boldsymbol{p} = -\mathrm{e}\sum_l Z_l \boldsymbol{R}_l + \int \boldsymbol{r}\rho(\boldsymbol{r})d\boldsymbol{r}$, where e is the electron charge, the l summation is over Mo and S ionic sites, and $\rho(\boldsymbol{r})$ is the electronic charge density in the MoS_2 interlayer. Both VB and CB of the intact MoS_2 were perturbed by some Au states, as depicted in (**a**), which indicates Au states electronically hybridize with states of MoS_2. We plotted local density of states (LDOS) of the interface in **(d)** in order to reveal the interactions between MoS_2 and Au (111). Only the Gamma point was used for clarity. Grey and red lines represent the LDOSs of an interfacial S atom (denote S_B) and an interfacial Au atom (denote Au_T). The hybrid states of S_B and Au_T were marked by black and light blue arrows. Each of those peaks contains six states while some of those states were representatively visualized in **(e)** to **(h)**. Panels **(e)** and **(f)** plot the isosurface contours of wavefunction norms for the bonding and anti-bonding states sitting at ~ -5.3 eV with an isosurface value of 1×10^{-4} e $Bohr^{-3}$, while those for the states at ~ -7.0 eV were shown in **(g)** and **(h)** with an isosurface value of 3×10^{-5} e $Bohr^{-3}$,

respectively. The hybridization between them is, however, not as strong as a typical covalent bond. The energy splitting of bonding-states and anti-bonding states is only few meV for those states marked by the black arrows and is up to 86 meV for those states marked by the light-blue arrows. While both the bonding and anti-bonding states all fully occupied, together with the rather small energy splitting, we conclude the interaction between Au and MoS_2 is not covalent bonding, but an interaction type called covalent-like quasi-bonding, as we discussed in the main text and recently identified in 2D layers.

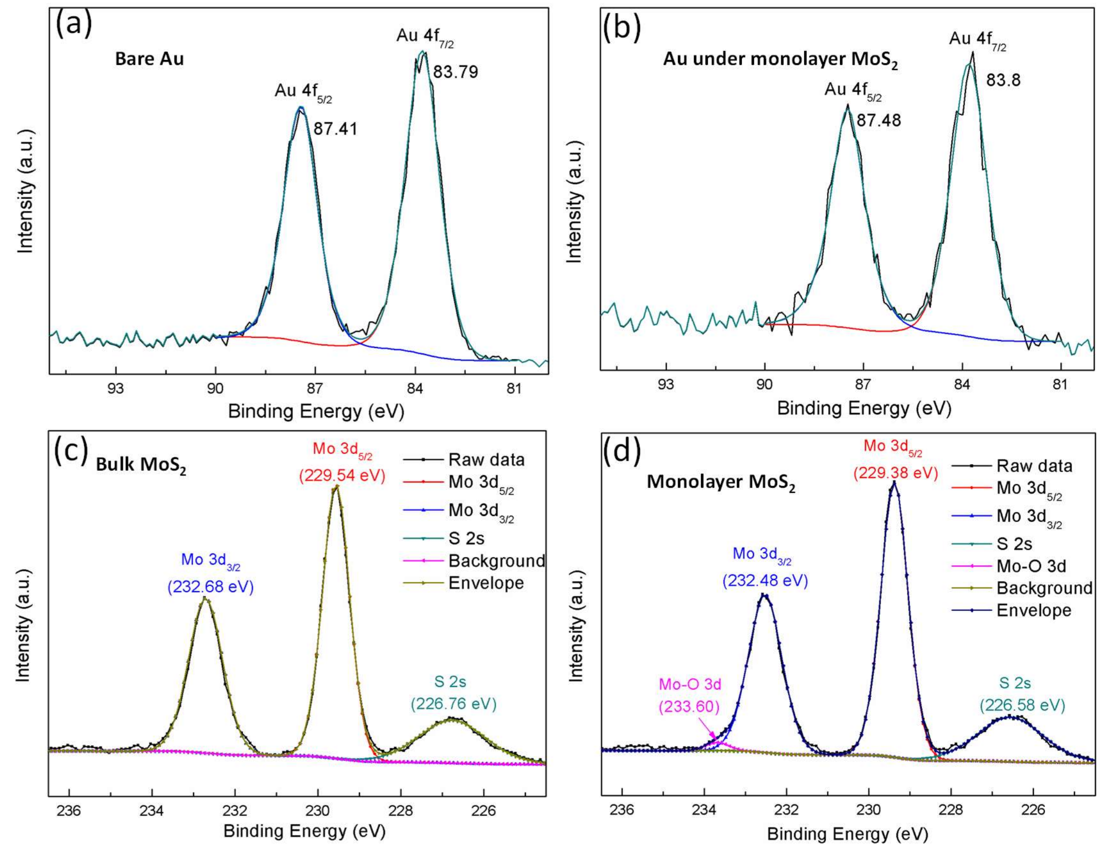

Supplementary Figure 2. XPS analysis was employed to obtain the core-level XPS spectra for the comparison of exfoliated single layer MoS_2 and bulk MoS_2 flakes. **(a, b)** XPS spectra of Au 4f from bare Au surface and the Au beneath a monolayer MoS_2 flake. They do not show noticeable difference of the binding energies but with a slightly decreased intensity for the Au spectra under MoS_2 flake due to the screening effect. **(c, d)** Mo 3*d* and S 2*s* spectra and the corresponding fitting curves of a bulk MoS_2 flake and of an exfoliated single layer MoS_2 flake. The core-level Mo 3*d* and S 2*s* spectra of the exfoliated MoS_2 show a small redshift (around 0.2 eV) when compared with the bulk MoS_2 bulk flakes, suggesting a possible charge transfer between the single layer MoS_2 flake and the underlying Au film[3, 4]. In addition, the consistence of the shape and peak width of the Mo 3*d* spectra of the exfoliated single layer MoS_2 with the bulk MoS_2 flake indicates that the single layer MoS_2 is clean and retains its chemical identity after exfoliation. This XPS result demonstrates the important role of Au for the successful exfoliation of large single layer MoS_2 flake.

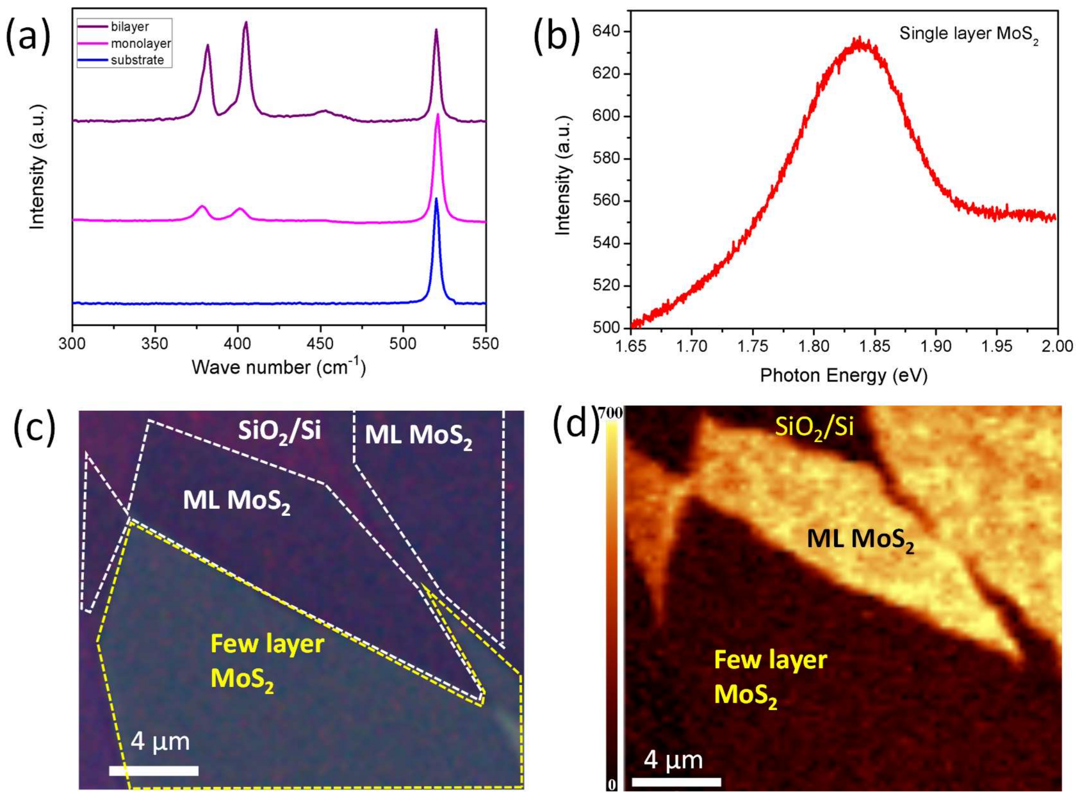

Supplementary Figure 3. **Raman and PL spectra of MoS_2 exfoliated with Au/Ti adhesion layer (nominally 1.5 nm/0.5 nm). (a)** Raman spectra of monolayer and bilayer MoS_2, normalized by the intensity of the Si peak at 520 cm^{-1}. **(b)** PL spectrum of monolayer MoS_2 exfoliated onto the ultrathin Au/Ti adhesion layer. **(c)** Optical image of a MoS_2 flake with coexisting monolayer and few-layer areas. **(d)** PL mapping image of the MoS_2 flake shown in **(c)**, with PL intensity in the monolayer area substantially higher than in few-layer and substrate regions. Raman peaks of E^1_{2g} and A_{1g} are at 386 and 406 cm^{-1}, respectively. From the Raman result, we can determine the following: first, the space between the two peaks (Δ) is ~20 cm^{-1}, suggesting that the as-exfoliated material is monolayer; second, the Raman peaks show no split, suggesting that the crystal quality of the as-grown MoS_2 film is good. The PL peak of A exciton is at 1.83 eV. These features are in good agreement with the data seen previously for monolayer MoS_2.

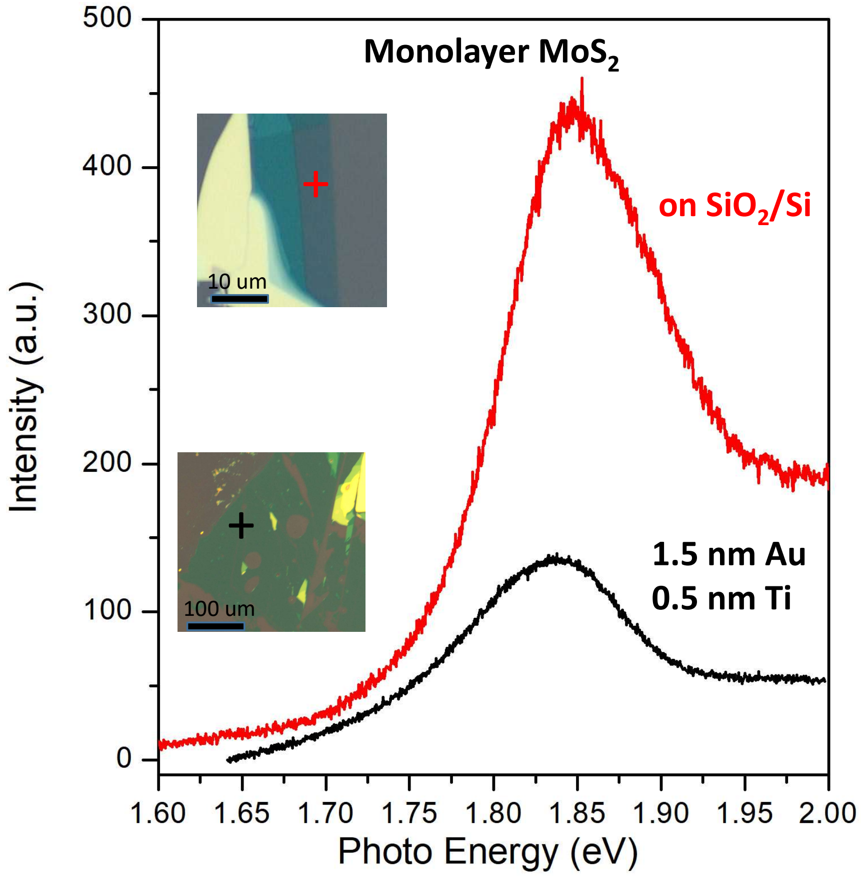

Supplementary Figure 4. Comparison of PL spectra between MoS_2 monolayers exfoliated on the SiO_2/Si substrate using the common tape method (red curve) and on the Au/Ti film using the present gold-assisted method (black curve), respectively.

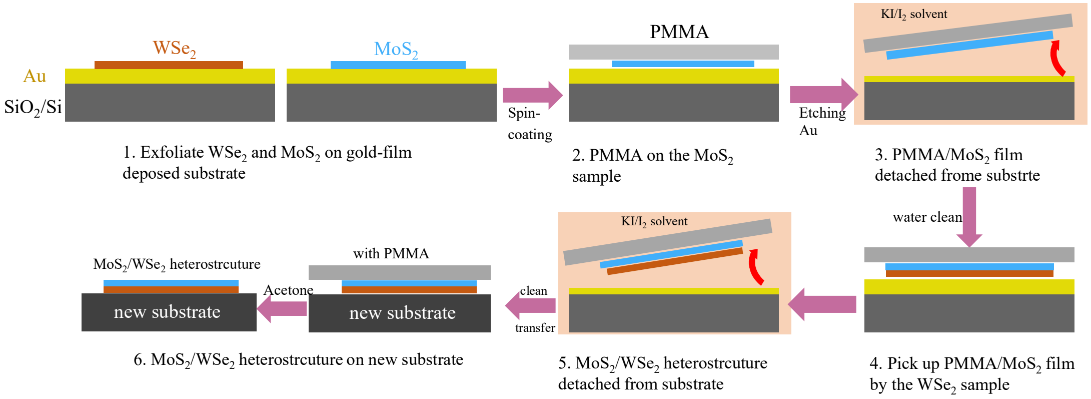

Supplementary Figure 5. Fabrication process of the MoS_2/WSe_2 heterostructure. Firstly, we exfoliated large area MoS_2 and WSe_2 monolayers on two separate $Au/Ti/SiO_2/Si$ substrates, respectively. Then, PMMA was spin-coated onto the MoS_2 monolayer and then the sample was put into KI/I_2 solution. After roughly 10 hours of etching, the gold film was removed and the PMMA film together with the MoS_2 flake detached from the SiO_2/Si substrate. In order to clean the ion residual, the PMMA film was washed three times using DI water. The next step lies in using the WSe_2 sample to pick up the PMMA/MoS_2 film from water. Since both MoS_2 and WSe_2 flakes are several millimeters in size, no special alignment is need in this step if the twisting angle between these two layers is not specified. Additional baking at ~ 100 °C ensures the contact between these two layers. Next, we employed the same procedure, i.e. etching in KI/I_2 solution and three times washing using DI water, to remove the Au film from WSe_2. A new substrate, e.g. SiO_2/Si was used to pick up the hetero-bilayer from Di water. Finally, we removed the PMMA layer using acetone.

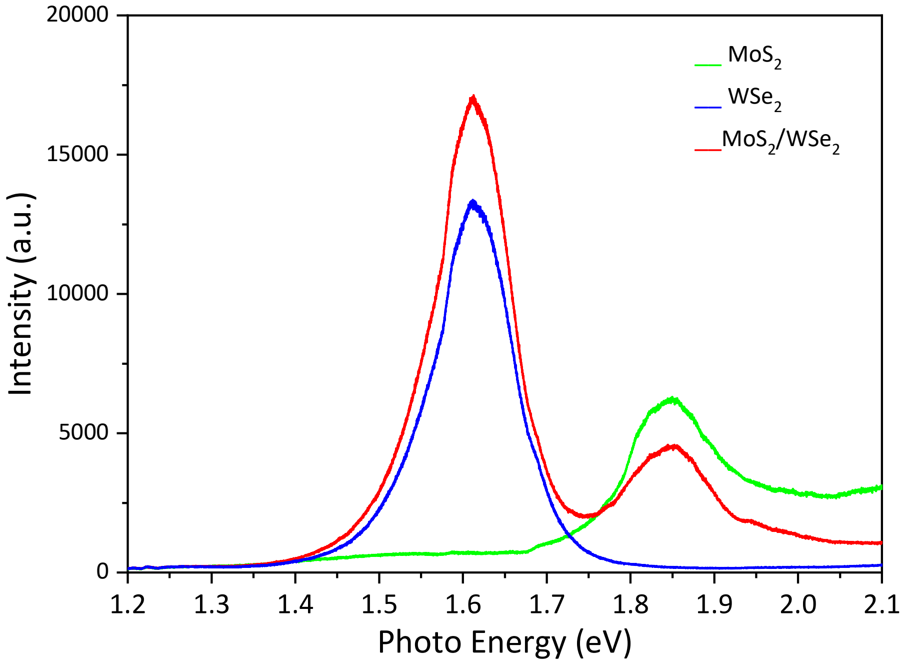

Supplementary Figure 6. PL spectra of MoS_2/WSe_2 heterostructure.

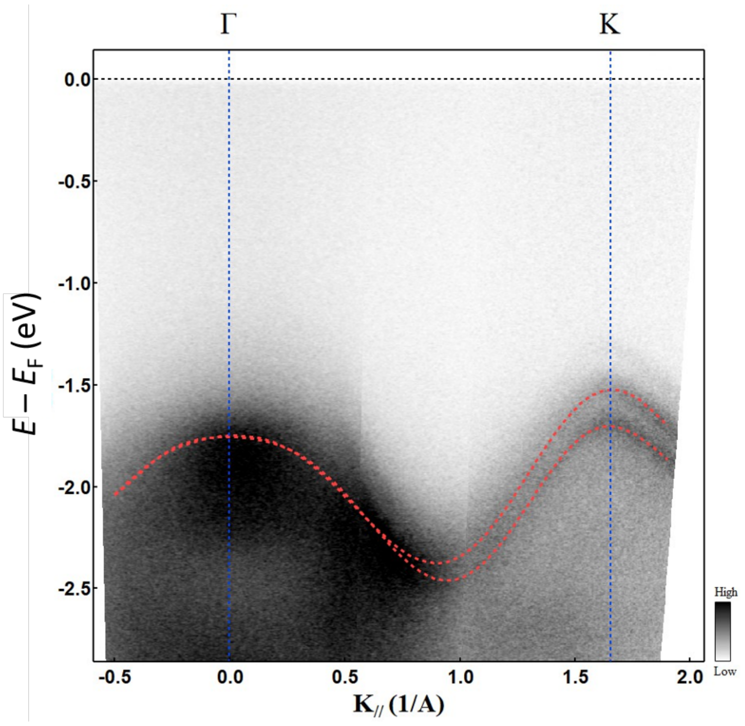

Supplementary Figure 7. Band structure of monolayer MoS_2 flake exfoliated on Au/Ti adhesion layer (5 nm/1 nm).

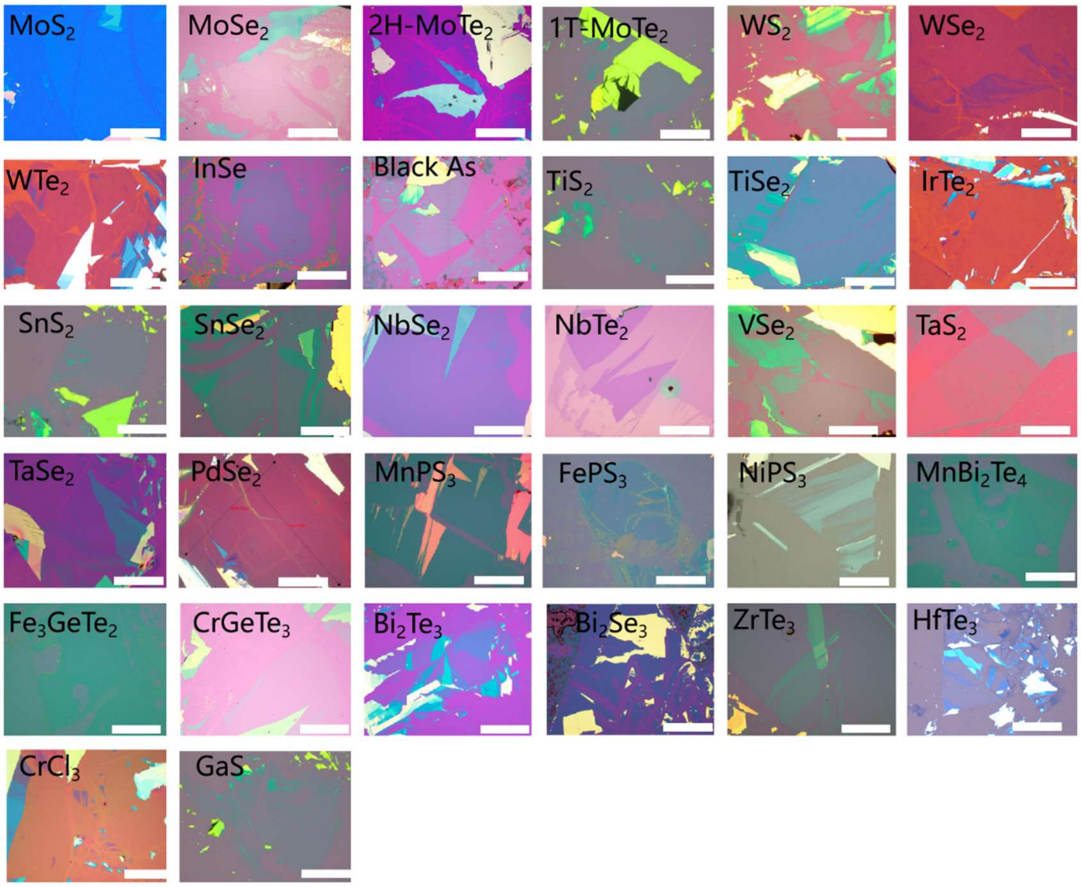

Supplementary Figure 8. Optical images of 2D materials exfoliated using Au adhesion layers. Some of materials shown are obtained here for the first time in monolayer form, for example, $IrTe_2$, $ZrTe_3$, $PtSe_2$. Scale bars: 500 μm.

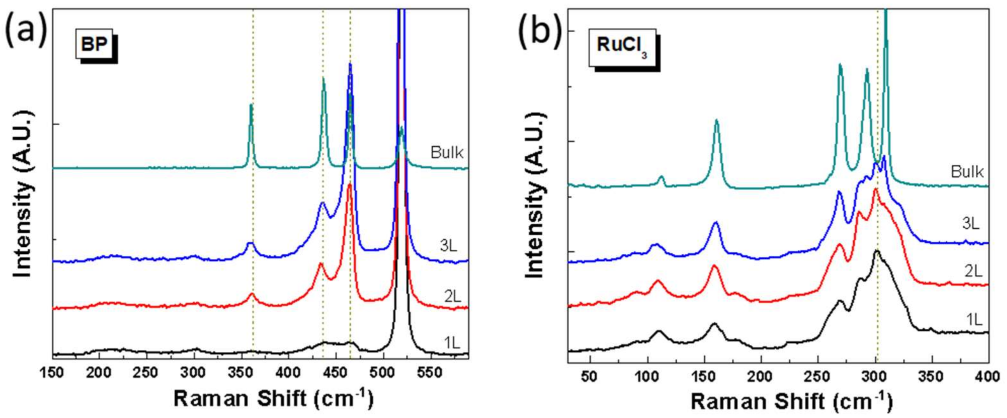

Supplementary Figure 9. Raman spectra of exfoliated black phosphorus (BP) **(a)** and $RuCl_3$ **(b)** crystals with different thickness, in comparison with the corresponding layered bulk crystals. In close analogy to bulk, three typical Raman peaks are resolved at 360, 436, and 465 cm^{-1} for the monolayer and few-layer BP samples shown in panel **(a)**. It can be seen that the phonon intensity decreases with layer numbers. The evolution of Raman intensity versus the number of layers is attributed to the multilayer interference occurring for both the incident light and the emitted Raman radiation, being akin to the case of graphene[5] and MoS_2[6, 7]. As for monolayer and few-layer α-$RuCl_3$ samples, five strong and sharp phonon modes are resolved at 117, 164, 270, 296 and 312 cm^{-1}, respectively, in panel **(b)**. It should be noted that the two lowest energy phonons in α-$RuCl_3$ show asymmetric Fano line shape stemmed from the coupling between the discrete optical phonons and the magnetic scattering, as we have discussed before[8]. For all thicknesses, it can be seen that the energies of all phonons are independent on the number of layers, as indicated by the dashed vertical lines. This is in marked contrast to TMDC[9] and indicates that the van der Waals interlayer interactions in BP and α-$RuCl_3$ are extremely weak and have tiny effects on phonon energy.

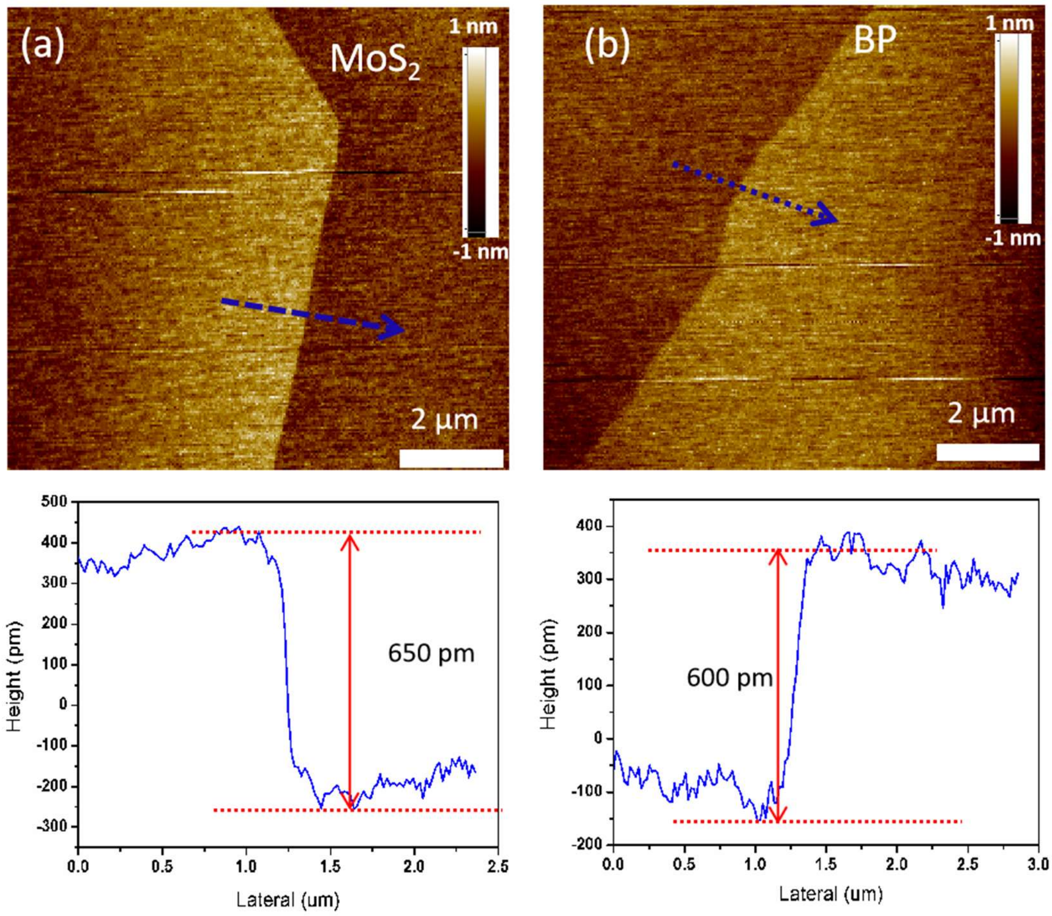

Supplementary Figure 10. AFM images of as-exfoliated 2D MoS_2 **(a)** and black phosphorus/phosphorene (BP) **(b)**. Given the the morphology of the freshly exfoliated samples at the nanometer scale, there is no evidence of structural irregularity or bubbles on the surfaces. The height profiles taken along the blue lines in AFM images are depicted in the lower parts of panels **(a)** and **(b)**. It is noted that the heights of monolayer MoS_2 and BP on the SiO_2/Si substrate are ~0.65 nm and ~0.60 nm, respectively, slightly larger than their theoretical thicknesses. Such deviation might imply that there are some absorbents at the interface between the MoS_2 or BP layer and the SiO_2/Si substrate[6, 10].

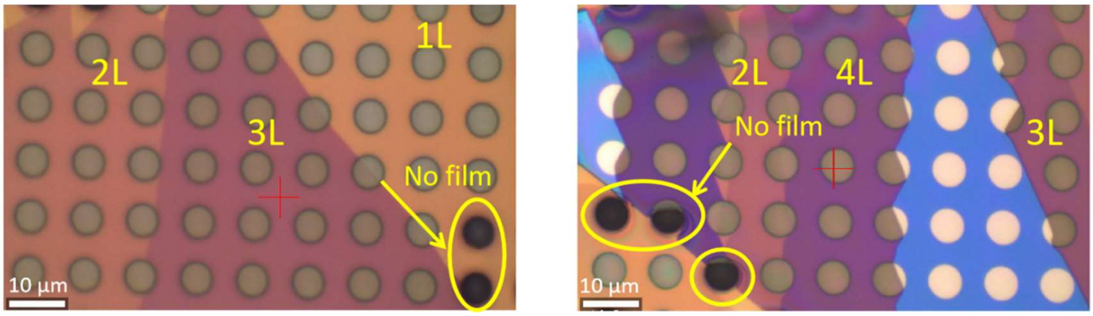

Supplementary Figure 11. Suspended WSe_2 samples with suspended coverage of 97% and 93%, respectively.

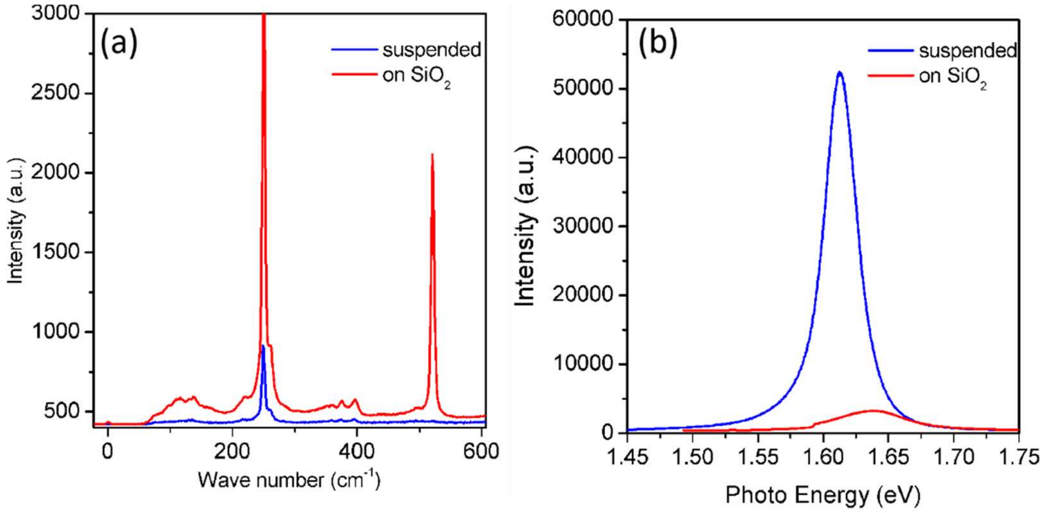

Supplementary Figure 12. Raman **(a)** and PL **(b)** spectra of suspended monolayer WSe_2 (blue) in comparison with monolayer WSe_2 exfoliated on a SiO_2/Si substrate (red), which explicitly show a roughly 10 times enhancement of PL intensity for suspended samples.

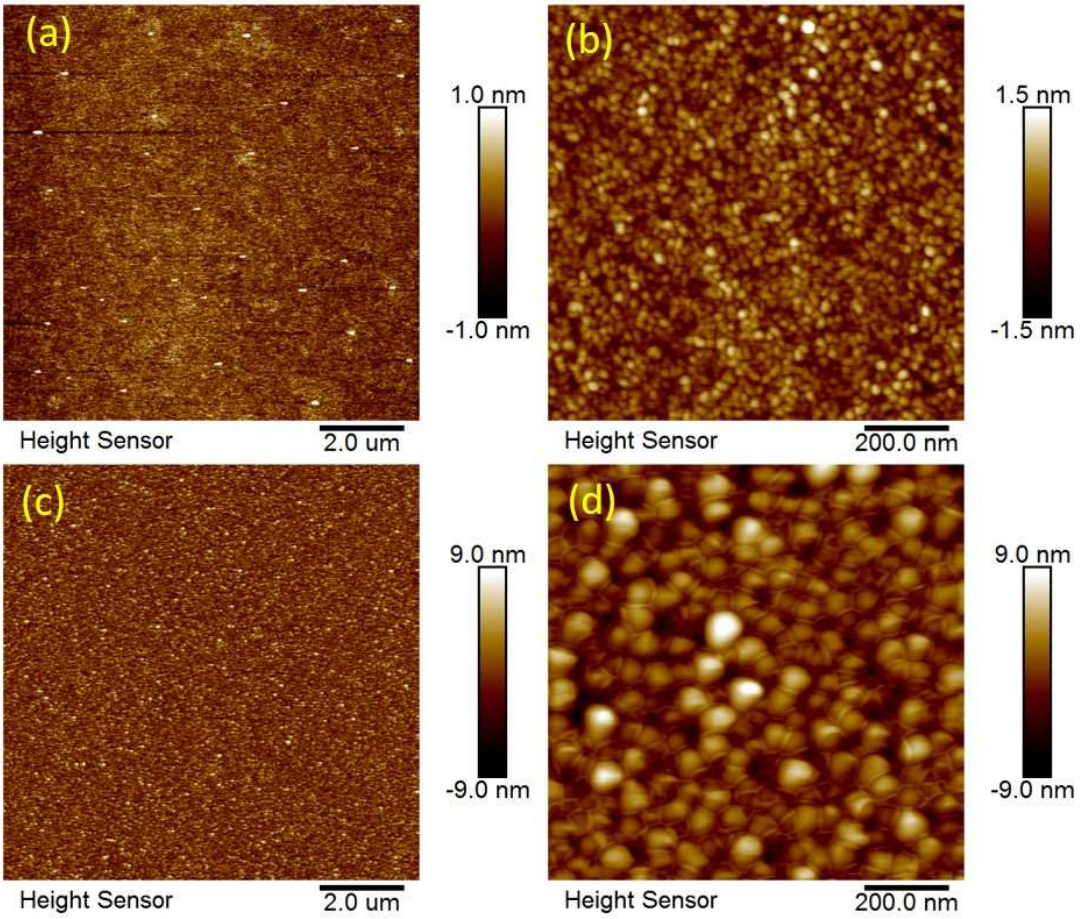

Supplementary Figure 13. AFM images of Au/Ti (1.5 nm/ 0.5 nm) film before (**a** and **b**) and after **(c,d)**. annealing at 250 ^{0}C for 2h. **(a)** and **(b)** are large-scale and zoom-in AFM images of an as-prepared metal thin-film sample, respectively. The size of metal grains appreciably increases after annealing, as shown in panels **(c)** and **(d)**. These AFM images demonstrate that the metals (Au/Ti) deposited on SiO_2/Si substrate is not a continuous film but some metal clusters with boundaries, which can become more obvious after annealing. Therefore, the substrate surface is still not conductive even an ultrathin metal layer was deposited on it.

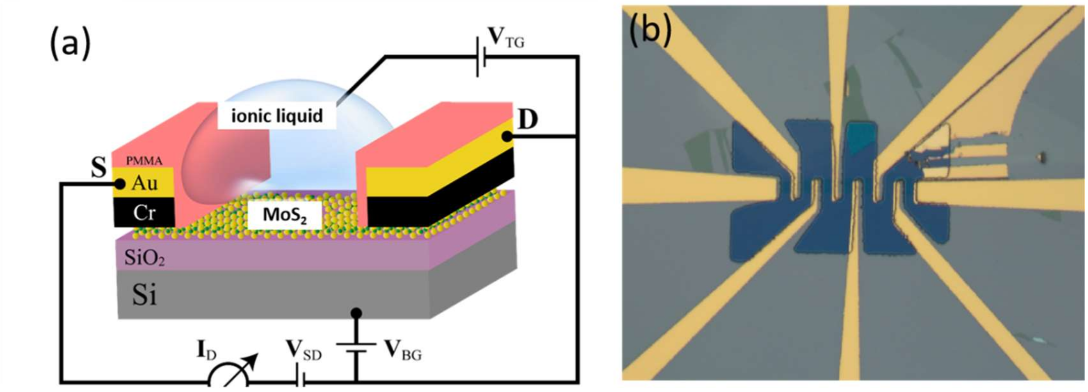

Supplementary Figure 14. (a) Monolayer field-effect transistor (FET) device structure with ionic liquid top gate. **(b)** Optical micrograph of an actual FET device with monolayer MoS_2 channel, and an insulating PMMA coating with window for the ionic liquid top gate.

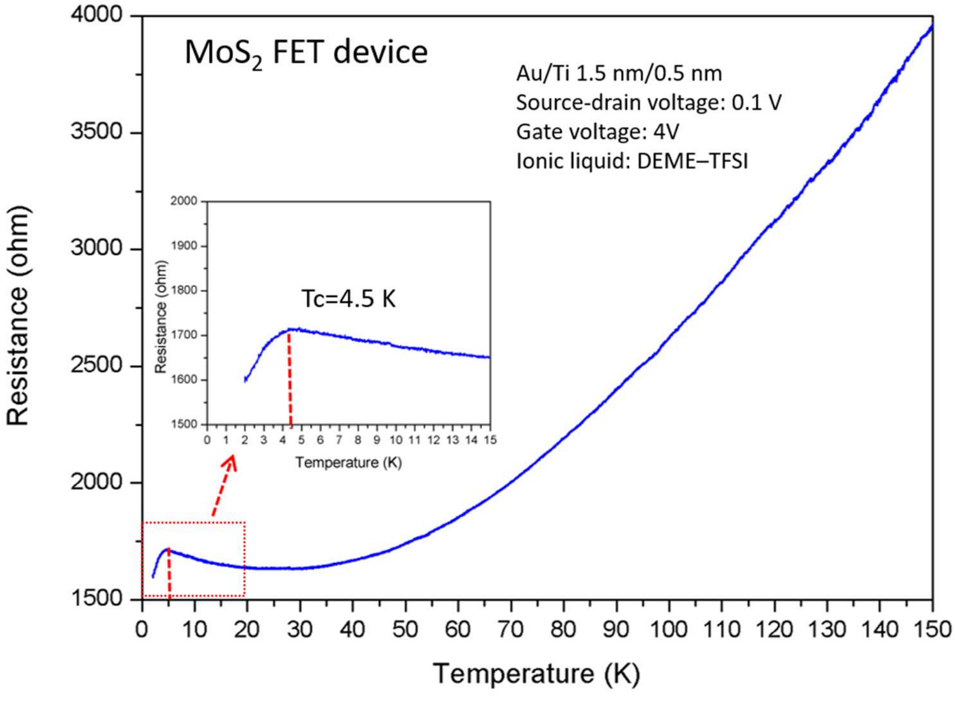

Supplementary Figure 15. Temperature dependent *I-V* curve of a monolayer MoS_2 device gated by ionic liquid (DEME-TFSI) at 4 V. The measurement temperature ranges from 2 K to 150 K. The inset is a zoom-in I-V curve at temperature from 2 to 15 K, from which a superconducting transition is indicated at T_c=4.5 K.

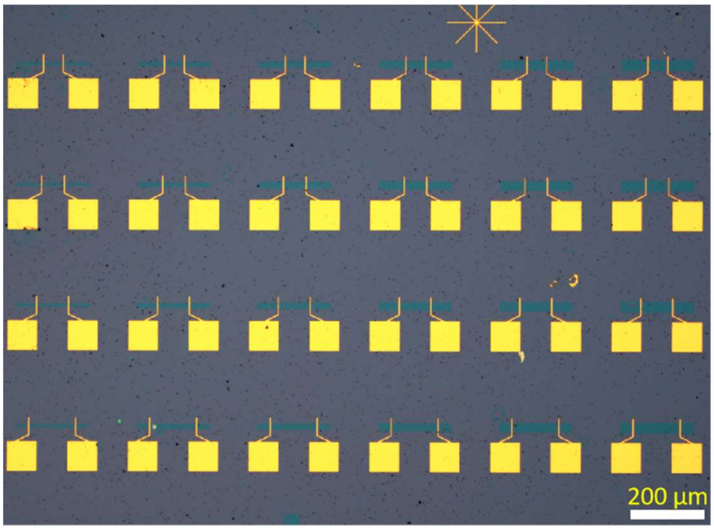

Supplementary Figure 16. Optical image of UV-patterned MoS_2 device arrays fabricated from a single large monolayer MoS_2 flake exfoliated onto an ultrathin Au/Ti adhesion layer.

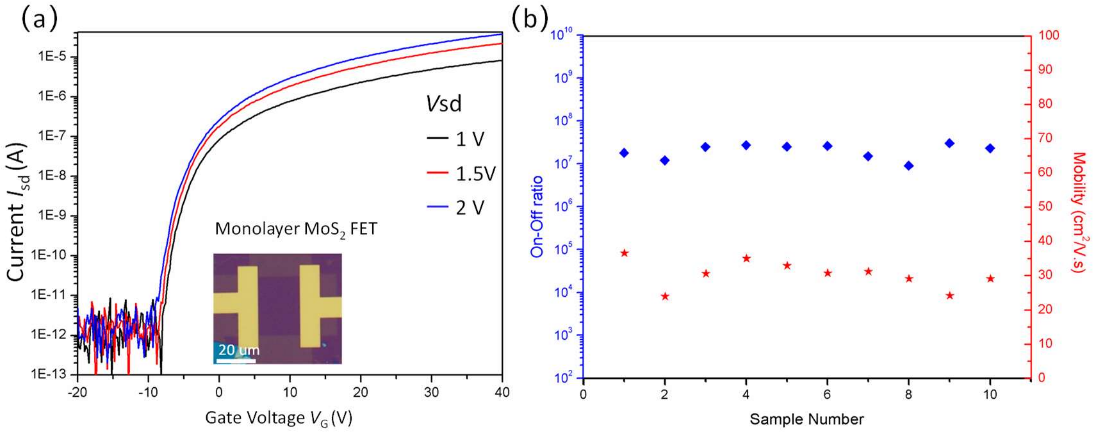

Supplementary Figure 17. Electrical measurements of monolayer MoS_2 back-gated FET devices. **(a)** Relationship between source-drain current I_{sd} and back gate voltage V_g for source-drain voltage V_{sd} ranging from 1 to 2 V. **(b)** Statistics of on-off ratio (blue) and mobility (red) of ten monolayer MoS_2 back-gated FET devices. The monolayer MoS_2 flakes were transferred to Si wafer (with 300 nm SiO_2 layer) by etching gold film in KI/I_2 solvent and DI water cleaning, as demonstrated in Supplementary Fig. 5. Then, the FET devices were fabricated by patterning, metal deposition and lift-off procedures.

Supplementary Tables

Supplementary Table 1. Calculated energies of all 58 considered 2D materials

2D-Materials	Ads. Energy on Au(111) (eV per unit cell)	Interlayer Coupling Energy (eV per unit cell)	Ads. Energy on Au(111) (eV Å^{-2})	Interlayer Coupling Energy (eV Å^{-2})	Ads. Energy on Au(111) (eV per bottom atom)	Interlayer Coupling Energy (eV per bottom atom)	$R_{LA/IL}$	Space Group	Magnetic Structure*
h-BN	0.146	0.136	0.0268	0.0250	0.073	0.068	1.07	P -6 m 2	NM
Gr	0.147	0.118	0.0279	0.0225	0.073	0.059	1.24	P 63/m m c	NM
P(Black)	0.801	0.484	0.0556	0.0334	0.400	0.242	1.67	C m c a	NM
As(Black)	1.014	0.584	0.0597	0.0350	0.507	0.292	1.71	C m c a	NM
W_2N_3	0.520	0.230	0.0716	0.0316	0.520	0.230	2.26	P 63/m m c	NM
TiS_2	1.087	0.542	0.0547	0.0273	0.543	0.271	2.00	P -3 m 1	NM
VS_2	0.412	0.265	0.0476	0.0305	0.412	0.265	1.56	P -3 m 1	FM
NbS_2	0.605	0.264	0.0632	0.0276	0.605	0.264	2.29	R 3 m H	NM
TaS_2-2H	0.549	0.249	0.0575	0.0262	0.549	0.249	2.19	P 63/m m c	NM
TaS_2-1T	0.457	0.278	0.0469	0.0286	0.457	0.278	1.64	P -3 m 1	NM
MoS_2	0.345	0.229	0.0397	0.0263	0.345	0.229	1.51	P 63/m m c	NM
WS_2	0.329	0.227	0.0371	0.0261	0.329	0.227	1.42	P 63/m m c	NM
ReS_2	1.128	0.856	0.0312	0.0211	0.282	0.214	1.48	P -1	NM
$CoPS_3$	4.883	1.433	0.0829	0.0211	1.221	0.358	3.35	C1 2/m 1	FM
$NiPS_3$	1.946	1.354	0.0336	0.0233	0.486	0.339	1.44	C1 2/m 1	FM
PdS_2	1.479	0.889	0.0494	0.0290	0.739	0.445	1.71	P b c a	NM
PtS_2	0.457	0.292	0.0421	0.0262	0.457	0.292	1.60	P -3 m 1	NM
GaS	0.388	0.218	0.0347	0.0196	0.194	0.109	1.78	P 63/m m c	NM
InS	1.093	1.025	0.0718	0.0588	0.547	0.512	1.22	P m n n	NM
GeS_2	0.316	0.273	0.0262	0.0223	0.316	0.273	1.17	P 42/n m c Z	NM
SnS	0.90	0.617	0.0534	0.0365	0.451	0.309	1.46	P n m a	NM
SnS_2	0.488	0.243	0.0418	0.0207	0.488	0.243	2.02	P -3 m 1	NM
$TiSe_2$	0.483	0.289	0.0454	0.0270	0.483	0.289	1.68	P -3 m 1	NM
VSe_2	0.476	0.261	0.0502	0.0272	0.476	0.261	1.85	P -3 m 1	FM
$NbSe_2$	0.607	0.304	0.0590	0.0295	0.607	0.304	2.00	P 63/m m c	NM
$MoSe_2$	0.414	0.243	0.0441	0.0258	0.414	0.243	1.71	P 63/m m c	NM
WSe_2	0.384	0.241	0.0409	0.0256	0.384	0.241	1.60	P 63/m m c	NM
$ReSe_2$	1.458	0.906	0.0373	0.0232	0.364	0.227	1.61	P -1	NM
FeSe	0.565	0.381	0.0424	0.0285	0.565	0.381	1.49	C m m a	AFM
$PdSe_2$	1.284	1.117	0.0388	0.0288	0.642	0.559	1.34	P b c a	NM
$PtSe_2$	0.641	0.341	0.0537	0.0276	0.641	0.341	1.95	P -3 m 1	NM
GaSe	0.352	0.228	0.0285	0.0185	0.176	0.114	1.54	P 63/m m c	NM
InSe	0.519	0.267	0.0369	0.0188	0.259	0.133	1.97	P 63/m m c	NM
$GeSe_2$	0.411	0.335	0.0304	0.0242	0.411	0.335	1.25	P 42/n m c Z	NM
SnSe	1.033	0.658	0.0560	0.0353	0.516	0.329	1.58	P n m a	NM
$SnSe_2$	0.621	0.275	0.0488	0.0214	0.621	0.275	2.24	P -3 m 1	NM

Sb_2Se_3	0.630	0.340	0.0448	0.0239	0.630	0.340	1.88	R -3 m H	NM
Bi_2Se_3	0.618	0.336	0.0416	0.0224	0.618	0.336	1.86	R -3 m H	NM
$ZrTe_3$	1.283	0.633	0.0554	0.0274	0.428	0.211	2.02	P1 21m/m 1	NM
$ZrTe_5$	2.378	1.088	0.0431	0.0186	1.189	0.544	2.32	C m c m	NM
$HfTe_3$	1.307	0.637	0.0567	0.0279	0.436	0.212	2.03	P1 21m/m 1	NM
$HfTe_5$	1.538	1.078	0.0280	0.0186	0.308	0.216	1.51	C m c m	NM
VTe_2	0.658	0.295	0.0585	0.0262	0.658	0.295	2.23	P -3 m 1	FM
$MoTe_2$	0.490	0.281	0.0456	0.0261	0.490	0.281	1.75	P 63/m m c	NM
WTe_2	1.026	0.516	0.0471	0.0235	0.513	0.258	2.00	P m n 21	NM
$MnBi_2Te_4$	0.791	0.368	0.0497	0.0229	0.791	0.368	2.17	R -3 m H	FM
FeTe	0.654	0.329	0.0461	0.0232	0.654	0.329	1.99	C m m a	AFM
$IrTe_2$	0.870	0.683	0.0680	0.0503	0.870	0.683	1.35	P -3 m 1	NM
$PtTe_2$	0.865	0.479	0.0631	0.0334	0.865	0.479	1.89	P -3 m 1	NM
Sb_2Te_3	0.773	0.404	0.0489	0.0256	0.773	0.404	1.91	R -3 m H	NM
Bi_2Te_3	0.842	0.397	0.0508	0.0235	0.842	0.397	2.16	R -3 m H	NM
$CrCl_3$	0.800	0.550	0.0300	0.0180	0.267	0.183	1.67	R -3 H	FM
$RuCl_3$	1.111	0.574	0.0356	0.0184	0.370	0.191	1.94	P 31 1 2	AFM
$CdCl_2$	0.260	0.203	0.0204	0.0158	0.260	0.203	1.29	R -3 m H	NM
$CrBr_3$	1.693	1.207	0.0245	0.0175	0.282	0.201	1.40	R -3 H	FM
$CdBr_2$	0.323	0.222	0.0234	0.0160	0.323	0.222	1.47	R -3 m H	NM
CrI_3	1.425	0.715	0.0344	0.0175	0.475	0.119	1.96	R -3 H	FM
CdI_2	0.387	0.247	0.0248	0.0157	0.387	0.247	1.57	R -3 m H	NM

*NM: Non-magnetic; FM: Ferromagnetic; AFM: Antiferromagnetic

Supplementary References

1. Chen, M.X. & Weinert, M. Layer k-projection and unfolding electronic bands at interfaces. *Physical Review B* **98**, 245421 (2018).
2. Chen, M.X., Chen, W., Zhang, Z.Y. & Weinert, M. Effects of magnetic dopants in ($Li_{0.8}M_{0.2}OH$)FeSe (M = Fe, Mn, Co): Density functional theory study using a band unfolding technique. *Physical Review B* **96**, 245111 (2017).
3. Desai, S.B. et al. Gold-Mediated Exfoliation of Ultralarge Optoelectronically-Perfect Monolayers. *Advanced Materials* **28**, 4053-4058 (2016).
4. Shi, J.P. et al. Monolayer MoS_2 Growth on Au Foils and On-Site Domain Boundary Imaging. *Advanced Functional Materials* **25**, 842-849 (2015).
5. Ferrari, A.C. et al. Raman spectrum of graphene and graphene layers. *Physical Review Letters* **97**, 4 (2006).
6. Lee, C. et al. Anomalous Lattice Vibrations of Single- and Few-Layer MoS_2. *ACS Nano* **4**, 2695-2700 (2010).
7. Zhang, H. et al. Interference effect on optical signals of monolayer MoS_2. *Applied Physics Letters* **107**, 4 (2015).
8. Du, L.J. et al. 2D proximate quantum spin liquid state in atomic-thin alpha-$RuCl_3$. *2D Materials* **6**, 8 (2019).
9. Deng, Y.J. et al. Gate-tunable room-temperature ferromagnetism in two-dimensional Fe_3GeTe_2. *Nature* **563**, 94 (2018).
10. Zhou, B.Y. et al. Possible structural transformation and enhanced magnetic fluctuations in exfoliated alpha-$RuCl_3$. *Journal of Physics and Chemistry of Solids* **128**, 291-295 (2019).
