# Supplementary material for: Universal mechanical exfoliation of large-area 2D crystals
Source: Nat Commun. 2020 May 15;11:2453. doi: 10.1038/s41467-020-16266-w (PMC7228924; doi:10.1038/s41467-020-16266-w)
Supplement: Supplementary file 2 — Description of Additional Supplementary Files [file 41467_2020_16266_MOESM2_ESM.docx]

**Description of Additional Supplementary Files**

**File name:** Supplementary Movie 1

**Description:** Video illustrating the exfoliation process
